# Effectiveness and usability of the system for assessment and intervention of frailty for community-dwelling pre-frail older adults: A pilot study

**DOI:** 10.3389/fmed.2022.955785

**Published:** 2022-11-17

**Authors:** Ren Siang Tan, Eileen Fabia Goh, Di Wang, Robin Chung Leung Chan, Zhiwei Zeng, Audrey Yeo, Kalene Pek, Joanne Kua, Wei Chin Wong, Zhiqi Shen, Wee Shiong Lim

**Affiliations:** ^1^Institute of Geriatrics and Active Ageing, Tan Tock Seng Hospital, Singapore, Singapore; ^2^Joint NTU-UBC Research Centre of Excellence in Active Living for the Elderly, Nanyang Technological University, Singapore, Singapore; ^3^Department of Geriatric Medicine, Tan Tock Seng Hospital, Singapore, Singapore; ^4^Lee Kong Chian School of Medicine, Nanyang Technological University, Singapore, Singapore; ^5^School of Computer Science and Engineering, Nanyang Technological University, Singapore, Singapore

**Keywords:** frailty, pre-frail, older adults, community research, health technology, intervention, usability

## Abstract

**Background:**

Effective multicomponent interventions in the community targeted at preventing frailty in at-risk older adults can promote healthy ageing. However, there is a lack of studies exploring the effectiveness of technology-enabled autonomous multi-domain community-based interventions for frailty. We developed a novel end-to-end System for Assessment and Intervention of Frailty (SAIF) with exercise, nutrition, and polypharmacy components. This pilot study aimed to explore SAIF’s effectiveness in improving frailty status, physical performance and strength, and its usability in pre-frail older adults.

**Materials and methods:**

This is a single arm 8-week pilot study in 20 community-dwelling older adults who were pre-frail, defined using the Clinical Frailty Scale (CFS) as CFS 3 + (CFS 3 and FRAIL positive) or CFS 4. For outcomes, we assessed frailty status using the modified Fried Frailty Phenotype (FFP) and CFS; physical performance using Short Physical Performance Battery (SPPB); and Hand Grip Strength (HGS) at baseline and 8-week. User experience was explored using the System Usability Scale (SUS), interest-enjoyment subscale of the Intrinsic Motivation Inventory and open-ended questions. We analyzed effectiveness using repeated-measures tests on pre-post scores, and usability using a convergent mixed-method approach *via* thematic analysis of open-ended responses and descriptive statistics of usability/interest-enjoyment scales.

**Results:**

Sixteen participants (71.8 ± 5.5 years) completed the 8-week study. There was a significant improvement in FFP score (−0.5, *p* < 0.05, effect size, *r* = 0.43), but not CFS (−1.0, *p* = 0.10, *r* = 0.29). Five (31.3%) improved in frailty status for both FFP and CFS. SPPB (+1.0, *p* < 0.05, *r* = 0.42) and HGS (+3.5, *p* < 0.05, *r* = 0.45) showed significant improvements. Three themes were identified: “Difficulty in module navigation” (barriers for SAIF interaction); “User engagement by gamification” (facilitators that encourage participation); and “Perceived benefits to physical health” (subjective improvements in physical well-being), which corroborated with SUS (68/100) and interest-enjoyment (3.9/5.0) scores. Taken together, user experience results cohere with the Senior Technology Acceptance and Adoption Model.

**Conclusion:**

Our pilot study provides preliminary evidence of the effectiveness of SAIF in improving frailty status, physical performance and strength of pre-frail older adults, and offers user experience insights to plan the follow-up large-scale randomized controlled trial.

## Introduction

Frailty is an aging-related syndrome characterized by diminished physiological reserves and an increased vulnerability to adverse health outcomes (1). According to the physical phenotype model of Fried et al., frailty may be identified by the presence of at least three of the following components: weakness, unintentional weight loss, slowness, exhaustion, and low physical activity (1). Research has shown that frailty is associated with an increased risk of falls, disability and mortality (1, 2). Frailty is also associated with a greater utilization of healthcare resources such as hospitalization (3) and emergency department visits (4). Healthcare professionals and authorities increasingly recognized frailty as a pressing public health priority due to its significant negative impact on the health of older adults and burden on healthcare systems (5, 6). Longitudinal studies show that the risk of transitioning to greater states of frailty increases with age (7, 8). However, as frailty is potentially reversible, there is increasing interest in shifting the focus toward early identification and timely intervention in the intermediate pre-frail stages (9, 10).

In recent years, emerging technological innovations have transformed the healthcare landscape by providing new opportunities to improve the management of chronic diseases for seniors (11, 12). Similarly, in the context of frailty, there is emerging evidence from small-scale studies supporting the benefit of technology-related intervention studies in pre-frail older adults. Recent studies reported that interventions leveraging sensor-based technology (e.g., video consoles or wearables) for exergaming (13–15) or walking programs (16) can improve frailty status and walking speed, and reduce falls risk in pre-frail seniors. In addition, exercise programs tapping upon telecommunications technology (*via* web or mobile applications) to provide remote instructional guides (17) or weekly telephone coaching (18) reported improvement in quality of life and functional performance in pre-frail older adults. Another trend is the rise in the exploration of Artificial Intelligence (AI) enabled technologies to track frailty progression and provide personalized interventions (19, 20). However, technological interventions tend to exist as isolated, single-domain solutions which do not harness a multi-component approach for interventions.

As a multi-dimensional syndrome with many etiological factors, a multi-domain approach is required to effectively manage the early stages of frailty in the community (6, 21). The Asia-Pacific Clinical Guidelines (21) for the management of frailty have outlined four evidence-based interventions: (i) physical exercise; (ii) polypharmacy reduction; (iii) caloric, protein and Vitamin D supplementation; and (iv) screening for reversible causes of fatigue. Using a combination of physical exercise, nutritional supplementation and cognitive training in a 6-month intervention for pre-frail and frail older persons, Ng et al. found significant improvements in frailty score and status in the multi-domain intervention group which persisted up to 12 months (10). Similarly, a primary-care based multi-component intervention in pre-frail and frail older persons was effective in reversing frailty measures at 18 months (22). Of note, a recent 12-week multi-component frailty prevention program for prefrail community-dwelling older persons reduced frailty and improved physical and cognitive functions and self-rated health (23). While the multi-component intervention studies demonstrated effectiveness in frailty outcomes, they are often resource-intensive with reliance on trained personnel to run the programs, which may affect the scalability and sustainability of these programs.

Against this backdrop, AI-enabled technology-based innovations provide a viable solution to render multi-component interventions in a scalable manner to alleviate the resource burden. Yet, the effective use of technology to deliver multi-component frailty interventions has hitherto been understudied. Older adults have greater variability in digital literacy as compared to their younger counterparts (24), and past studies have highlighted that some older adults may face barriers to digital adoption due to low perceived ease of use (25–27). Thus, we propose a multi-domain, end-to-end System for Assessment and Intervention of Frailty (SAIF), which aims deliver a holistic user-friendly intervention for frailty in the community. SAIF leverages data-driven AI technologies to provide individualized polypharmacy management, nutritional recommendation, and physical exercises. In this pilot study, we aim to explore the effectiveness of SAIF in improving frailty status, physical performance and strength in community-dwelling pre-frail older adults who are at risk of frailty progression. We also aim to explore the usability of SAIF in terms of adherence, safety and user experience.

## Materials and methods

### Study design and participants

The study design was an 8-week single arm, pre-post pilot trial. Data was collected at baseline and the end of the 8-week SAIF intervention. Participants were recruited from Peace-Connect Cluster Operator (PeCCO), a senior activity center in Singapore. SAIF intervention sessions and study visits were conducted at PeCCO. Participants were screened using the eligibility criteria stated in the next section and eligible participants were enrolled into the study. Written informed consent was obtained from all participants before their participation in this study. Ethics approval of the study was obtained from the Domain Specific Review Board of the National Healthcare Group (Ref: 2019/01217). This pilot study has not been registered. However, the main study for “An End-to-end System for Assessment and Intervention of Frailty (SAIF)” is registered under clinicaltrials.gov (NCT05371210).

### Eligibility criteria

Participants fulfilling all of the following inclusion criteria were eligible for the study: (1) aged 60 years old and above; (2) speak English and/or Mandarin; (3) able to ambulate more than 10 m without walking aid; and (4) pre-frail, defined using the Clinical Frailty Scale (CFS) (28, 29) as either: (i) CFS 3 + [CFS 3 with positive item(s) endorsed on FRAIL scale (30)], or (ii) CFS 4. Participants exhibiting at least one of the following criteria were excluded from the study: (1) Has Dementia/Parkinson’s Disease/Arthritis; (2) hip surgery within the last 6 months; (3) hospitalized within the last 1 month; (4) presence of end-stage organ failure/symptomatic heart conditions/Chronic Obstructive Pulmonary Disease; and (5) modified Chinese Mini-Mental State Examination (CMMSE) < 19 (31).

### System for assessment and intervention of frailty intervention

**FIGURE 1:**
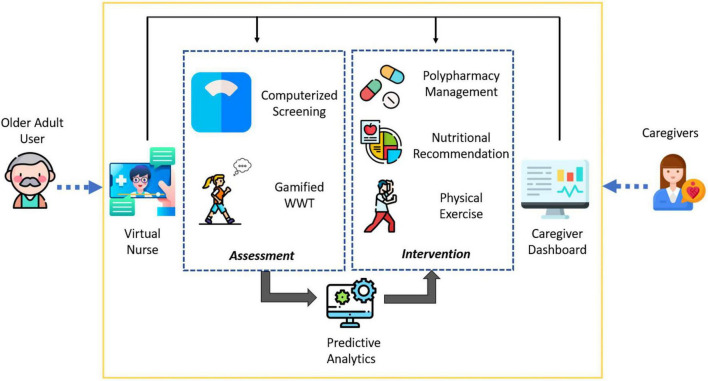
Conceptual diagram of system for assessment and intervention of frailty (SAIF) depicting the integrated elements of interface, assessment, and intervention components.

The technical aspects of SAIF have been described in detail elsewhere (32; Figure 1). In the design phase of the system, clinical inputs were sought from healthcare professionals to develop the interventional modules. Human factors such as familiarity and elderly-friendly human-machine interaction techniques were adopted to ensure that SAIF was simple and intuitive for use by older adults. Designed as an autonomous interventional system, older adults may utilize SAIF with minimal supervision or assistance from caregivers. Before the start of SAIF interaction, the system developers engaged participants one-to-one and demonstrated its usage before allowing the older adults to try on their own. Automated instructions, available in both Chinese and English, are embedded in the system together with a voice-over to help guide the older adult user for subsequent sessions. Additionally, the system development team stationed staff members in the first couple of weeks of the study to ensure that the older adults were able to interact with SAIF independently and correctly and provided remote support afterward whenever technical issues arose. Instructional manuals were also provided to community center staff members to help assist subjects whenever necessary.

**FIGURE 2:**
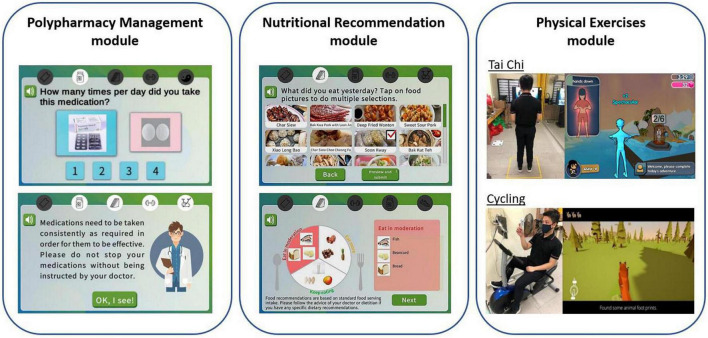
Illustration of system for assessment and intervention of frailty (SAIF) intervention modules which consist of polypharmacy management, nutritional recommendation, and physical exercises modules.

Three interconnected elements form the SAIF architecture: (1) interface modules (Virtual Nurse and Caregiver Dashboard) which act as the medium for users to interact with the system; (2) assessment modules to collect subjective questionnaire responses and objective physical measurements and feed the input into a predictive analytics model for frailty status prediction, and (3) intervention modules aimed at managing frailty through a multi-domain approach. For the purpose of this paper, we focus on the intervention modules in SAIF. In line with the Asia-Pacific Clinical Practice Guidelines for frailty management (21), SAIF interventional modules include polypharmacy management, nutritional recommendations and physical exercises (Figure 2).

#### Polypharmacy management module

The polypharmacy management module aims to help users track and improve medication adherence. Prior to the start of SAIF interaction, participants’ medication regimen was recorded by research assistants. Pictures of their medications (packaging box and/or pills) were also included to aid in questionnaire selection. Responses regarding medication adherence were collected through self-reported questionnaires administered on the tablet, where the Virtual Nurse resides. SAIF acted as an electronic monitoring system to display the correct frequency and dosing for participants who did not comply with their medication regimen. Remote reminders have been shown to be effective in improving medication adherence (33, 34). Information on medication effects were also delivered by the Virtual Nurse to encourage users to stay on track with their health goals. For users who stopped their medications, the system provided prompts to seek advice from their healthcare provider about the medications.

#### Nutritional recommendation module

The nutritional recommendation module aims to provide personalized dietary suggestions based on the analysis of the food intake information provided by the participants. Remote dietary assessment and tailored nutritional feedback has gained traction in the past few years for their potential in health promotion (35–37). Users selected their diet intake from a variety of food categories displayed on the tablet. Pictures of local food cuisines were included to aid the older adult users in food selection. Based on the calculated nutritional values of the selected foods, dietary modeling tools generated food recommendations to supplement nutritional needs. SAIF also harnessed data from the regular dietary inputs from users to identify individual preferences to personalize food recommendations.

To elaborate, the dietary intake upload module does not take into consideration personal information such as height, weight, and activity level. However, it considers other personal information which is provided by the participants, such as tabooed food items due to religious reasons, food allergy or personal preference. For instance, for a Muslim participant, all food items with pork or lard ingredients are filtered out in the food selection to input their diet intakes. Participants are prompted to upload all the food items consumed in the previous day whenever they logged into SAIF.

The food items are organized in a two-level manner which first selects the broad category (such as drink, fruit, type of meat, type of cuisine, etc.) and then the exact food item (such as apple, steak, spicy tofu, etc.). In this 2-month study, the order of both the higher-level categories and the lower-level food items were set by our team members based on heuristics.

#### Physical exercise module

Tai Chi and Cycling modules are exercise kiosks for training upper and lower limbs, respectively. Each exercise session lasted for 15 min, and on each day during the study period, each participant was randomly assigned by the Virtual Nurse to conduct one of the two exercises. Game elements were incorporated for the Tai Chi and Cycling programs as gamification (i.e., the usage of game elements in a non-game context) can improve the interest and engagement in physical activities for older adults (38) and motivate users to maintain their adherence to the program (39, 40).

Tai Chi is a prominent exercise derived from martial arts, and is popular with Chinese older adults (41). This form of exercise has been shown to improve the balance capacity of seniors (42). The SAIF Tai Chi exercise module is conducted *via* a large-screen TV mounted with Kinect-based motion sensors. Users were instructed to follow a series of upper-body Tai Chi moves demonstrated by a virtual coach as well as to perform a set of stretching exercises with resistance bands for warm-up, and a coin-catching mini game for warming down (32). The Kinect-based motion sensors detect body movements of participants and game points were awarded for accurate replication of the Tai Chi moves by participants.

Stationary cycling is a form of endurance training that can promote physical health in older adults and is relatively safe (43). The SAIF Cycling module consists of a stationary exercise bike with motion sensors mounted onto the pedals. A tablet is mounted onto a stand in front of the exercise bike for the game display. Users were represented by a virtual avatar in a hunting game where the avatar will move according to the pedal speed detected (32). Participants were tasked to race forward and track their prey to earn game points upon capturing the prey.

In line with the self-determination theory, features were incorporated to motivate and sustain user participation (44, 45). For instance, successful completion of the instructional tasks within the games elicited positive messages to commend and reinforce their behaviors. Points were accumulated as participants cleared different levels of the game. A game points ranking system within each game was also incorporated to create extrinsic motivation through competition between the participants.

#### Procedure

**FIGURE 3:**
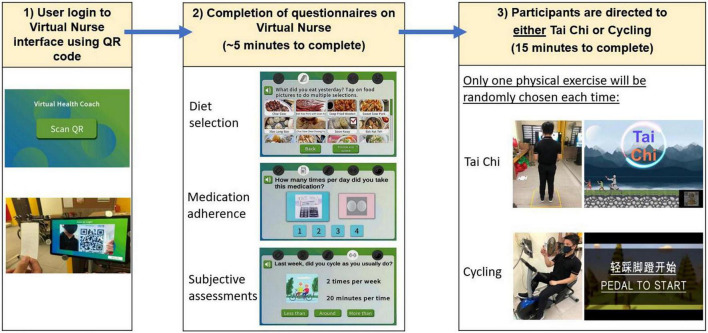
Overview of user interaction for system for assessment and intervention of frailty (SAIF) session: login to Virtual Nurse interface, followed by completion of diet, medication, and subjective assessment questionnaires, and then being directed to exercise module (either Tai Chi or cycling).

Prior to the start of the intervention, all participants were guided through one complete cycle of SAIF interaction (Figure 3). Participants accessed the Virtual Nurse interface using a personalized QR code. Upon login, they were asked to provide responses related to diet intake, medication adherence and subjective measures of frailty. Thereafter, they would be randomly directed to either the Tai Chi or Cycling exercise module. Successful completion of the questionnaires and assigned physical exercise were recorded as one interaction count. Each interaction session took approximately 20 min (5 min for questionnaires and 15 min for Physical Exercise module).

Community staff was trained to provide on-site technical assistance to users when they encountered difficulties. To ensure that effectiveness of the intervention was not undermined by inadequate adherence, participants were advised to complete at least 10 SAIF interactions in a month. Attendance was tracked electronically and authorized community staffs and study members monitored participants’ adherence *via* a web-based Caregiver Dashboard.

## Measurements

### Demographics and clinical assessments

Participants’ age, gender, race, education level, housing type, chronic conditions and number of medications were recorded. Standing height and body weight were measured to derive the body mass index (BMI). Cognitive performance was evaluated using the modified Chinese Mini-Mental State Examination (CMMSE) (31). Functional status was assessed using the modified Barthel Index for Basic Activities of Daily Living (BADL) (46) and Lawton’s scale for Instrumental Activities of Daily Living (IADL) (47).

### Outcome measures

#### Effectiveness

Physical performance and frailty status of participants were evaluated at baseline and 8-week. Physical performance was assessed using the Short Physical Performance Battery (SPPB) (48) and Hand Grip Strength (HGS). The SPPB is an objective measure of lower extremity functions (static balance, gait speed, and chair-stand) in older adults (48). The highest reading for HGS as measured twice on each hand using the North Coast™ Hydraulic Hand Dynamometer (North Coast Medical Inc., Morgan Hill, CA, USA) was recorded (49).

Frailty status was assessed using two measures: modified Fried Frailty Phenotype (FFP) and CFS. The modified FFP was calculated based on established values for Asian older adults (50) and operationalized using the following components: (1) Handgrip strength of < 28 kg for men and < 18 kg for women; (2) body mass index < 18.5; (3) usual gait speed < 1.0 m/s over the 3-meter walk test; (4) fatigue based on endorsement of either of two questions (“I felt that everything I did was an effort” and “I could not get “going””) from the Center for Epidemiologic Studies-Depression Scale (CES-D); and (5) low physical activity measured using the International Physical Activity Questionnaire – Elderly (IPAQ-E) and defined using cut-off of < 2,826 Metabolic Equivalent Task (MET) minutes per week (51). The CFS is a 9-point global rating scale which allows classification across the frailty continuum ranging from 1 (very fit) to 9 (terminally ill) (29). CFS 3 corresponds to non-frail individuals who are not regularly active beyond routine walking, while CFS 4 refers to the “vulnerable” group with symptoms of slowing or fatigue which limit activities (28). Frailty is diagnosed when individuals are categorized as CFS 5–8.

#### Usability

Adherence to the SAIF program was measured by the number of SAIF interventions completed. The criterion for success related to intervention adherence was for participants to comply with the instruction to attend a minimum of 10 sessions in a month. We also investigated the user safety of the system, as operationalized by the number of reported incidents out of the total interaction count.

User experience data was measured at the end of the 8-week intervention. For quantitative data, we utilized the System Usability Scale (SUS) which assesses the perceived usability of technological systems and includes 10 items scored on a 5-point Likert scale (0 = “strongly disagree” to 4 = “strongly agree”) (52). Item scores were added up and multiplied by 2.5 to obtain a score ranging between 0 and 100 (52). The SUS has been extensively used in previous user research studies and demonstrated good psychometric properties (52). Better usability was indicated by higher SUS scores (53). Interest-enjoyment subscale of the Intrinsic Motivation Inventory (54) was used as a self-report measure of interest and enjoyment of the intervention. The interest-enjoyment subscale comprised of seven items that were measured on a 5-point Likert scale (1 = “strongly disagree” to 5 = “strongly agree”) (54). The interest-enjoyment scores were calculated by averaging across the total number of items in the subscale.

Qualitative feedback was gathered using open-ended questions regarding the modules which participants liked the most/least and the reasons for their responses.

### Statistical analyses

Analyses were conducted on IBM SPSS version 26.0 (IBM Corporation, Armonk, NY, USA) with a two-tailed significance level of *p* < 0.05 considered as statistically significant. Continuous variables were expressed as means (standard deviation) or as medians (interquartile range). Categorical variables were expressed as counts (percentages).

Effectiveness was analyzed using Wilcoxon signed rank test on pre-and post-intervention scores for continuous variables. As we utilized a non-parametric analysis, correlational effect sizes (*r*) were computed (55) and interpreted according to Cohen’s guidelines (56): Small effect (*r* = 0.10), medium effect (*r* = 0.30), large effect (*r* = 0.50). The change in categorical frailty status between pre-and post-intervention time points was investigated using the McNemar’s test.

For user experience, we analyzed the quantitative and qualitative data separately using a convergent mixed-method approach (57) and the results were integrated *via* a joint display through visual means (58). Descriptive univariate analyses were performed on the SUS and interest-enjoyment scales. The qualitative responses collected were analyzed for themes using the thematic analysis framework by Braun and Clarke (59). Most Chinese participants had a good command of Mandarin and understood simple English. To ensure that they were able to answer to the best of their abilities, questions were asked in Chinese. The research assistant, who has proficiency in both English and Mandarin, transcribed the responses into written English. For Malay participants, both questions and responses collected were in English. For analysis, we followed the methods described by Bree and Gallagher (60) in using Microsoft Excel to aid in the generation of themes. Open codes were color-coded, categorized and grouped to form meaningful themes (61).

## Results

**FIGURE 4:**
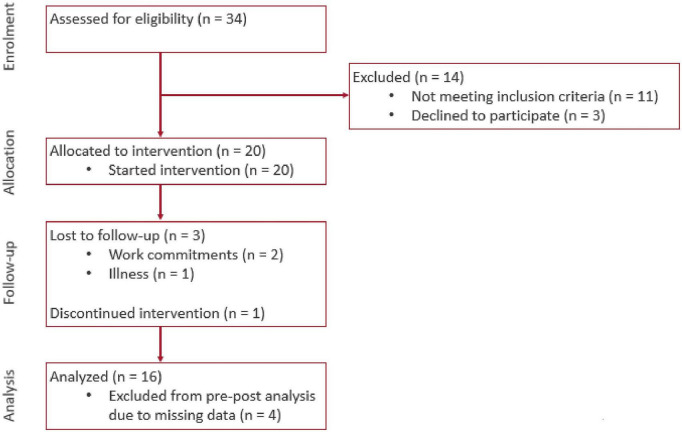
Consort flow diagram of progress through the phases of enrolment, allocation, follow-up, and data analysis.

Amongst 34 participants assessed for eligibility, we excluded 14 to yield a final sample of 20 participants (Figure 4). The mean age of participants was (70.9 ± 5.6 years). They were predominantly female (70%), of Chinese ethnicity (75%), and had either primary or secondary education (55 and 30% respectively) (Table 1). In terms of frailty status, most had a CFS score of 4 (70%) and the remaining (30%) were CFS 3 and pre-frail on the FRAIL scale. Correspondingly, the median BADL and IADL scores were 100 and 23 respectively (maximum scores), which attested to the relatively good health of the cohort. Out of the 20 recruited participants, 16 participants (71.8 ± 5.5 years) completed the 8-week intervention period. There was no significant difference in baseline characteristics such as age, gender, education, housing type, past medical history, CMMSE and functional status between dropouts and the remaining participants (Supplementary Table 1). Amongst the 4 participants who dropped out, 3 reported reasons unrelated to the intervention (2 had work commitments and 1 had illness). The last participant discontinued intervention due to difficulty navigating the Virtual Nurse module despite assistance being rendered to use the system.

**TABLE 1:** Baseline characteristics of study cohort.

Variables	Total (*n* = 20)
**Demographics**	
Age, years	70.9 ± 5.60
**Gender, n (%)**	
Female	14 (70.0)
Male	6 (30.0)
**Race, n (%)**	
Chinese	15 (75.0)
Malay	5 (25.0)
**Level of education, n (%)**	
No formal education	3 (15.0)
Primary	11 (55.0)
Secondary	6 (30.0)
Education, years	6.40 ± 3.58
**Housing, n (%)**	
1–2 room	19 (95.0)
3 room	1 (5.0)
**Medical history**	
Hypertension, n (%)	15 (75.0)
Hyperlipidemia, n (%)	17 (85.0)
Diabetes, n (%)	6 (30.0)
Asthma, n (%)	1 (5.0)
Cancer, n (%)	3 (15.0)
Current medications	4.30 ± 2.74
**Anthropometry**	
Weight, kg	61.38 ± 9.63
BMI, kg/m^2^	25.10 ± 4.02
**Cognitive performance**	
CMMSE, max 28	23.50 ± 2.37
**Functional status**	
BADL score*	100.00 (95.00–100.00)
IADL score*	23 (22–23)
**Frailty status**	
**FRAIL, n (%)**	
Robust	13 (65.0)
Pre-frail	7 (35.0)
Score*, max 5	0 (0–1.00)
**CFS, n (%)**	
CFS 3	6 (30.0)
CFS 4	14 (70.0)
Score*, (range 3–5)	4.00 (3.00–4.00)
**Physical performance**	
SPPB*, max 12	10.00 (8.00–11.00)
HGS, kg	16.35 ± 5.66

Mean ± SD unless otherwise indicated; *median (IQR); BADL, basic activities of daily living; BMI, body mass index; CFS, clinical frailty scale; CMMSE, Chinese mini-mental state examination; EQ–5D–5L, EuroQOL 5-dimension 5–level questionnaire; EQ-VAS, EuroQOL visual analogue scale; FFP, Fried frailty phenotype; HGS, hand-grip strength; IADL, instrumental activities of daily living; SPPB, short physical performance battery.

### Effectiveness

There was a significant improvement in modified FFP scores (Difference: −0.5, *p* < 0.05, *r* = 0.43) of moderate effect size, after the 8-week SAIF intervention. In terms of change in FFP frailty categories, five (31.3%) participants showed improvement (*p* = 0.063), with one improving from pre-frail to robust and four from frail to pre-frail. For CFS, there was a non-significant improvement (Difference: −1.0, *p* = 0.10, *r* = 0.29), and five participants improved from CFS 4 (pre-frail) to CFS 3 (non-frail sedentary) (*p* = 0.18). For physical performance, there was a significant improvement of moderate effect size in SPPB (+1.0, *p* < 0.05, *r* = 0.42); similarly, HGS showed a significantly improvement of moderate effect size (+3.5, *p* < 0.05, *r* = 0.45) (Table 2).

**TABLE 2:** Effectiveness outcome results (*n* = 16).

	Pre (baseline)	Post (8 weeks)	*P-value*	Effect size (*r*)
**Physical performance**				
SPPB, max 12	10.00 (7.25–11.75)	11.00 (10.00–12.00)	0.02^a^	0.42
Hand grip strength	16.50 (12.00–22.00)	20.00 (16.00–22.75)	0.01^a^	0.45
**Frailty status**				
FFP, max 5	2.50 (2.00–3.75)	2.00 (1.00–2.75)	0.02^a^	0.43
Robust	0 (0.0)	1 (6.3)	0.06^b^	
Pre-frail	8 (50.0)	11 (68.8)		
Frail	8 (50.0)	4 (25.0)		
CFS	4.00 (3.00–4.00)	3.00 (3.00–4.00)	0.10^a^	0.29
CFS 3	6 (37.5)	11 (68.8)	0.18^b^	
CFS 4	10 (62.5)	5 (31.3)		

^a^Wilcoxon signed rank test.

^b^McNemar’s test; two-tailed significance set at *p* < 0.05.

### Usability

The mean SUS score was 68.0 out of 100 (*SD* = 9.9), and the interest-enjoyment score was 3.9 out of 5.0 (*SD* = 0.4). The mean interaction count was 11.3 (81% attended 10 times or more) in the first month and 10.1 (63% attended 10 times or more) in the second month. Based on participants’ completion records over the period of 2 months, there was good adherence to the SAIF intervention as most participants were able to attend at least 10 sessions in a month. Out of the total participant interaction count of 363, there was 1 reported incident of participant feeling unwell during the course of SAIF interaction (0.3%).

### Themes

Three key themes emerged from thematic analysis of participant comments. They allude to the perceived benefits of the intervention to physical health, user engagement by gamification and difficulties encountered in navigating the modules.

#### Perceived benefits to physical health

Participants enjoyed the physical interventions as they noticed an improvement in their perceived physical health after the SAIF interaction. For instance, one participant noted that it “helped to improve leg strength” (P7). Similarly, another user shared that her “leg has become less painful, (and) can walk faster…” (P17).

#### User engagement by gamification

Gaming aspects utilized in the Tai Chi and Cycling modules motivated and engaged users. For instance, one user shared that she felt “motivated by the points” gained across the different levels of the games (P3). For another participant, the “money-catching segment (of the warm-down exercise for Tai Chi game) is fun… game scores also make it more fun and exciting” (P8). Other participants also commented that the games were interesting to them.

#### Difficulty in module navigation

Participants highlighted the barriers faced during interaction with SAIF. Some expressed difficulties with using the Virtual Nurse interface module. For example, participants highlighted difficulties in selecting the food options for the nutritional module. One participant found it “troublesome” that there were “too many things to press at food section” (P1) while another commented that she was “not sure how to use this function properly” (P3). For the Tai Chi physical exercise module, some participants noted that the Kinect may not be sensitive enough to detect user movements. To circumvent this, one participant shared how she “… needs to do bigger movements to score points” (P4).

### Mixed-methods integration

The Senior Technology Acceptance and Adoption Model (STAM) (62) was adapted to integrate the quantitative and qualitative results. STAM has been used in studies to explain the uptake and utilization of new technologies by older adults (63, 64) by alluding to procedural phases which older adults undergo during the process of technology uptake. In the incorporation phase, the older adult user explores and forms a perception about the technological system. Through this experimentation, positive experiences coupled with a perception of ease of use play an important role in the acceptance of technology for older adults (62).

**FIGURE 5:**
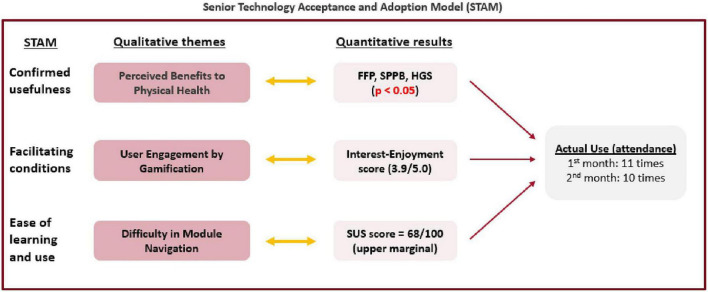
Joint-display of qualitative themes and quantitative results using the senior technology acceptance and adoption model (STAM).

The themes from our qualitative user feedback cohere with the incorporation phase as described in the STAM (Figure 5). Participants showed good adherence to SAIF system, as evidenced by the complementarity between the qualitative themes with the results of quantitative analysis. According to the STAM, confirmed usefulness and facilitating conditions are factors that can influence the actual use of the technological system. Through interaction with SAIF, older adult users perceived benefits to their physical health (Theme 1), which was corroborated by the significant improvements (*p* < 0.05) in their frailty status and physical performance. Gamification was a facilitating condition for participants (Theme 2), as they were engaged by the games, and this was reflected in the high interest-enjoyment score of 3.9 out of 5.0. Some participants faced difficulty during the module navigation (Theme 3), which may have contributed to the SUS score of 68 out of 100. Nonetheless, the perceived usefulness of SAIF and engagement from game elements facilitated good adoption from our participants, as reflected in the high usage of the system.

## Discussion

The World Health Organization proposed reframing the concept of healthy ageing to emphasize intrinsic capacity, which is a key determinant of an individual’s functional abilities (65). Intrinsic capacity and frailty are complementary concepts such that community-based multi-component interventions targeted at the prevention of frailty can inform strategies for healthy aging at the population level (66). Our pilot study contributes to the growing body of evidence which supports early intervention for frailty by investigating the effectiveness and usability of the 8-week SAIF program in community-dwelling pre-frail older adults. Results support the effectiveness of SAIF in improving frailty status, physical performance and strength. The participants also found SAIF to be interesting and enjoyable with fairly good usability. Taken together, our results support the notion of pushing the boundaries of frailty interventions to include the at-risk groups of CFS 3 to 4 before the onset of established frailty. Our pilot study has notable strengths. To the best of our knowledge, this is the first study to demonstrate the effectiveness and usability of a community-based unsupervised multi-domain technology-based intervention consisting of medication management, nutritional recommendation and physical exercises. The novelty of SAIF lies in the use of AI-driven technological applications to deliver an autonomous multi-domain frailty intervention.

The demonstration of effectiveness in terms of frailty status, physical performance and strength corroborates the results of recent intervention studies in cohorts consisting entirely of pre-frail older adults. Notable differences are worth highlighting. Firstly, the criteria to define frailty in prior studies included FFP (67) and FRAIL (23, 68); in contrast, we primarily based our inclusion criteria on CFS, which was supplemented with FRAIL in the CFS 3 (non-frail) group to select an enriched at-risk population. The strength of CFS resides in its simplicity as a measure of global frailty which has been validated in various populations in real-world settings (69). Secondly, approaches to frailty interventions in prior studies comprised either multi-component exercises (67) or interventions (23, 68) in multiple domains of exercise, nutrition, cognition and/or social. Our study likewise employed an evidence-based multicomponent approach premised on the Asia-Pacific Clinical Practice Guidelines for frailty management (21) which addressed the domains of exercise, nutrition and polypharmacy. Lastly, frailty outcome measures in prior studies comprise single measure of FFP or FRAIL. In comparison, the combination of FFP and CFS outcome measures in our study yielded complementary insights respectively about physical frailty (premised on the phenotypic approach) (1) and global frailty (premised on the deficit accumulation approach) (29, 70). The significant benefit in physical frailty is likely mediated *via* the positive intermediary effects on SPPB and grip strength, which in turn may be related to our physical exercise modules with both upper-(Tai Chi) and lower-(Cycling) body exercises. Tai Chi has been found to improve the balance of older adults (42), and aerobic exercises like stationary cycling were beneficial in improving seniors’ gait speed and chair-stand tests (71). These improvements in physical components, coupled with nutritional advice and medication management, may have led to the improvement in overall frailty, as demonstrated by the improvement in CFS status of some participants from pre-frail to non-frail, albeit with a larger sample size needed to demonstrate statistical significance with CFS.

User experience with SAIF demonstrated that older adults do not need complex digital skills to use the system as they were able to successfully complete the sessions independently. However, some participants found the food selection of the nutritional module to be complex and troublesome. The Kinect-motion sensors may also not be sensitive enough to detect some user movements. Our study thus highlights the importance of a comprehensive program evaluation in the pilot phase that also includes usability aspects of technological innovations such that insights from user experiences can inform improvements for the subsequent validation study (72). Future developments of the SAIF system can explore the usage of voice inputs, which showed good usability, accuracy and time efficiency for diet reporting amongst older adult users (73). Food categories and items could also be re-ordered according to their frequency of being uploaded by the participants (i.e., the display order following their popularity). Kinect sensitivity may also be improved with identification of optimal placement relative to participants’ standing distance (74). The low reported incident count (0.3%) highlighted that the system can be safely deployed in a community setting as an autonomous intervention for pre-frail older adults. Nonetheless, remote monitoring tools may be used to enhance the safety of users during physical exercises (75). Consequently, trigger mechanisms may be incorporated to activate immediate assistance for older adults (76).

We acknowledge several limitations. Firstly, the pragmatic single-group pre-post design in our exploratory study precludes definitive conclusions about causality. Specifically, the lack of a control group, the small sample size and multiple outcome measures being analyzed raise the possibility that the significant improvement in frailty, physical performance and strength could be the result of a Type-I error; conversely, the lack of significance for CFS outcomes could represent a Type-II error from an inadequately powered study. Secondly, we also did not specifically assess for the effects of mitigating factors such as boredom and mood on user compliance and the usability and interest-enjoyment scores. To study the effects of these factors, the user experience questionnaires may be collected at two time points (e.g., at the 1-month and 2-month follow-up respectively) along with in-depth qualitative interviews to better ascertain the impact of factors affecting motivation/compliance on the participant’s perceived levels of interest and enjoyment toward the intervention. Next, the lack of randomization and recruitment from a single center also meant that individuals who joined the research may not be representative of the older adult population in Singapore. Although the baseline CMMSE mean score of our participants is comparable to those reported in an earlier study of community dwelling older adults of normal cognition around the same age group (31), previous studies have highlighted the importance of considering demographic factors such as age, gender, ethnicity and educational level which can influence the level of technological literacy and adherence to health-related technological interventions (77, 78). As such, caution must be exercised in generalizing the effectiveness and usability of SAIF to other groups of older adults. Also, the multi-domain intervention of SAIF focuses on the 3 domains of nutrition, physical exercise and polypharmacy, and did not consider other domains such as sleep, psychological well-being and social interactions. Lastly, as SAIF is a multi-component intervention, it is difficult to gauge the independent contributions of the modules toward improving the physical health of our participants. However, it is also plausible that the modules may create a synergistic effect in improving the frailty status of our older adult subjects.

The current pilot study is preliminary and it sets the stage for a multi-centered, adequately powered, and randomized controlled study which incorporates an adequate duration of post-intervention follow-up assessments to ascertain the sustainability of the benefits in reducing frailty and improving physical performance and strength over time. Importantly, insights from usability data in the pilot study can inform improvements in the implementation and user experience of the SAIF system for the validation study.

## Conclusion

Frailty is a syndrome that may be prevented or reversed through appropriate interventions. Effective multi-component interventions in the community targeted at the prevention of frailty can inform strategies to promote healthy ageing at the population level. Our pilot study provides preliminary results to support the use of SAIF as an autonomous interventional system to reduce frailty and improve physical performance and strength in pre-frail older adults. SAIF’s novelty lies in the use of technology to deliver a data-driven, community-based, multi-component intervention to improve outcomes in pre-frail older adults. The implementation of a follow-up large-scale randomized controlled trial is warranted.

## Data availability statement

The datasets presented in this article are not readily available because, subject to institutional rules for data sharing. Requests to access the datasets should be directed to WL, Wee_Shiong_Lim@ttsh.com.sg.

## Ethics statement

The studies involving human participants were reviewed and approved by National Healthcare Group IRB. Written informed consent to participate in this study was obtained from study participants. In addition, written informed consent was obtained from the individual for the publication of any potentially identifiable images or data included in this article.

## Author contributions

RT was responsible for data analysis and writing the manuscript. WL was involved in study design, supervised the data analysis, and the critical appraisal of the manuscript. Rest of co-authors was involved in study design and critical appraisal of the manuscript. All authors contributed to the article and approved the submitted version.
