# Corrigendum: Effectiveness and usability of the system for assessment and intervention of frailty for community-dwelling pre-frail older adults: A pilot study

**DOI:** 10.3389/fmed.2022.1105448

**Published:** 2022-12-22

**Authors:** Ren Siang Tan, Eileen Fabia Goh, Di Wang, Robin Chung Leung Chan, Zhiwei Zeng, Audrey Yeo, Kalene Pek, Joanne Kua, Wei Chin Wong, Zhiqi Shen, Wee Shiong Lim

**Affiliations:** ^1^Institute of Geriatrics and Active Ageing, Tan Tock Seng Hospital, Singapore, Singapore; ^2^Joint NTU-UBC Research Centre of Excellence in Active Living for the Elderly, Nanyang Technological University, Singapore, Singapore; ^3^Department of Geriatric Medicine, Tan Tock Seng Hospital, Singapore, Singapore; ^4^Lee Kong Chian School of Medicine, Nanyang Technological University, Singapore, Singapore; ^5^School of Computer Science and Engineering, Nanyang Technological University, Singapore, Singapore

**Keywords:** frailty, pre-frail, older adults, community research, health technology, intervention, usability

In the published article, there were errors in the order of Figures 1–5 as published. The corrected Figures 1–5 and its captions appear below.

**Figure 1:**
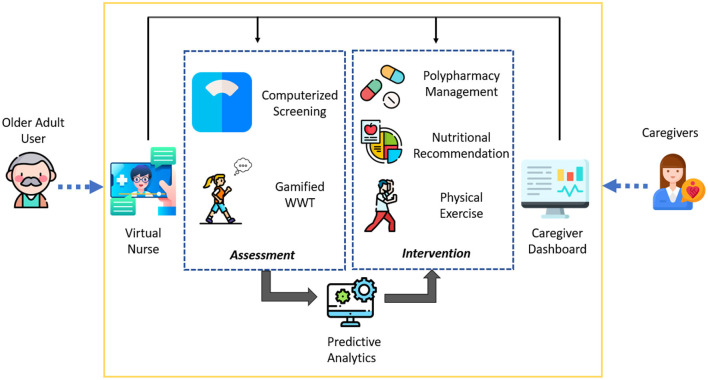
Conceptual diagram of system for assessment and intervention of frailty (SAIF) depicting the integrated elements of interface, assessment, and intervention components.

**Figure 2:**
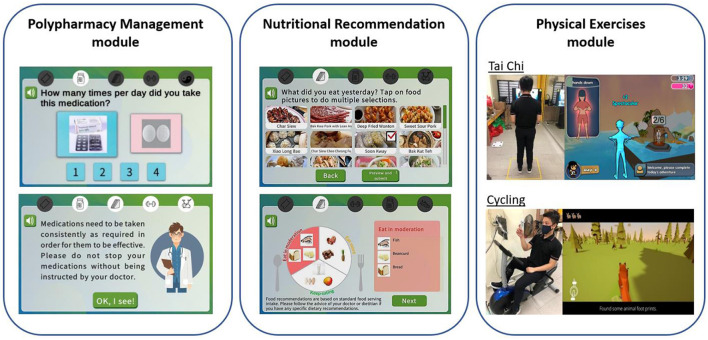
Illustration of system for assessment and intervention of frailty (SAIF) intervention modules which consist of polypharmacy management, nutritional recommendation, and physical exercises modules.

**Figure 3:**
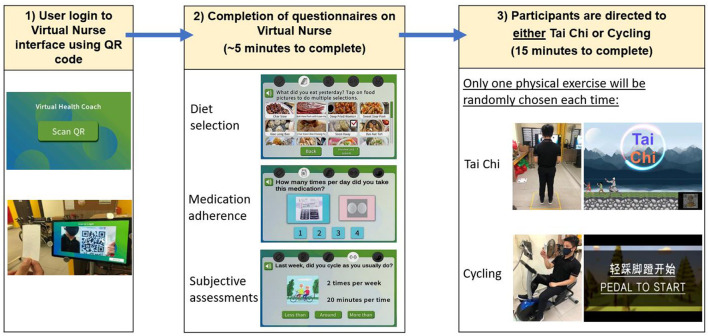
Overview of user interaction for system for assessment and intervention of frailty (SAIF) session: login to Virtual Nurse interface, followed by completion of diet, medication, and subjective assessment questionnaires, and then being directed to exercise module (either Tai Chi or cycling).

**Figure 4:**
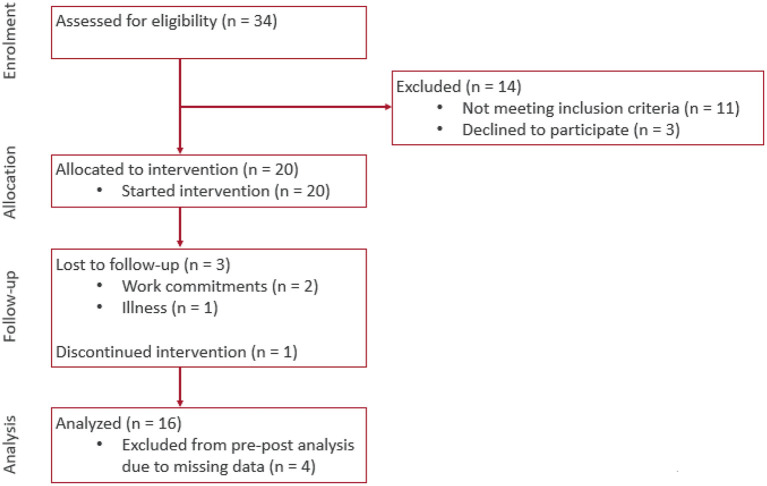
Consort flow diagram of progress through the phases of enrolment, allocation, follow-up, and data analysis.

**Figure 5:**
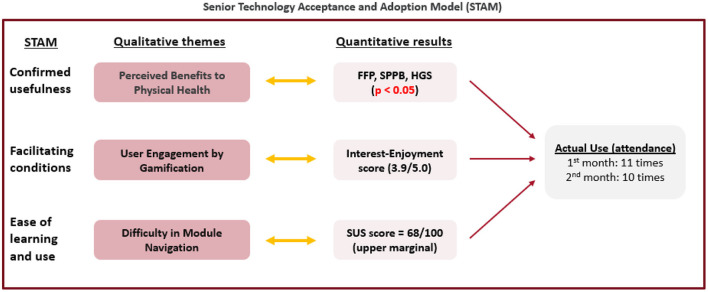
Joint-display of qualitative themes and quantitative results using the senior technology acceptance and adoption model (STAM).

In the published article, Figures 2, 3 contain identifiable images and the written informed consent obtained was not clearly stated in the **Ethics statement**.

A correction has been made to the **Ethics statement** section, paragraph 1. This sentence previously stated:

“The studies involving human participants were reviewed and approved by National Healthcare Group IRB. The patients/participants provided their written informed consent to participate in this study.”

The corrected sentence appears below:

“The studies involving human participants were reviewed and approved by National Healthcare Group IRB. Written informed consent to participate in this study was obtained from study participants. In addition, written informed consent was obtained from the individual for the publication of any potentially identifiable images or data included in this article.”

The authors apologize for this error and state that this does not change the scientific conclusions of the article in any way. The original article has been updated.

