# Supplementary material for: Effectiveness and usability of the system for assessment and intervention of frailty for community-dwelling pre-frail older adults: A pilot study
Source: Front Med (Lausanne). 2022 Nov 17;9:955785. doi: 10.3389/fmed.2022.955785 (PMC9713022; doi:10.3389/fmed.2022.955785)
Supplement: Supplementary file 1 [file Table_1.pdf]

Supplementary Table 1. Comparison of baseline characteristics between participants who completed study and drop-outs

Variables	Participants who completed study (n = 16)	Drop-outs (n = 4)	*p*-value
**Demographics**			
Age, years	71.75 ± 5.47	67.5 ± 5.45	0.18[a]
Gender, n (%)			
Female	11 (68.8)	3 (75.0)	0.81[b]
Male	5 (31.3)	1 (25.0)	
Race, n (%)			
Chinese	12 (75.0)	3 (75.0)	1.00[b]
Malay	4 (25.0)	1 (25.0)	
Level of education, n (%)			
No formal education	2 (12.5)	1 (25.0)	0.40[b]
Primary	10 (62.5)	1 (25.0)	
Secondary	4 (25.0)	2 (50.0)	
Education, years	6.50 ± 3.37	6.00 ± 4.90	1.00[a]
Housing, n (%)			
1 - 2 room	15 (93.8)	4 (100.0)	0.61[b]
3 room	1 (6.3)	0 (0.0)	
**Medical history**			
Hypertension, n (%)	12 (75.0)	3 (75.0)	1.00[b]
Hyperlipidemia, n (%)	14 (87.5)	3 (75.0)	0.53[b]
Diabetes, n (%)	5 (31.3)	1 (25.0)	0.81[b]
Asthma, n (%)	1 (6.3)	0 (0.0)	0.61[b]
Cancer, n (%)	3 (18.8)	0 (0.0)	0.35[b]
Current medications	4.25 ± 2.89	4.50 ± 2.38	0.63[a]
**Anthropometry**			
Weight, kg	60.95 ± 9.58	63.08 ± 11.08	0.70[a]
BMI, kg/m^2	25.13 ± 4.21	24.94 ± 3.74	0.93[a]
**Cognitive performance**			
CMMSE, max 28	23.44 ± 2.34	23.50 ± 2.89	1.00[a]
**Functional status**			
BADL score*	100.00 (95.00 – 100.00)	100.00 (96.25 – 100.00)	0.73[a]
IADL score*	23 (22 – 23)	23 (22.25 – 23.00)	0.73[a]
**Frailty status**			
FRAIL, n (%)			
Robust	10 (62.5)	3 (75.0)	0.64[b]
Pre-frail	6 (37.5)	1 (25.0)	
Score*, max 5	0 (0 – 1.00)	0 (0 – 0.75)	0.58[a]
CFS, n (%)			
CFS 3	6 (37.5)	0 (0.0)	0.14[b]
CFS 4	10 (62.5)	4 (100.0)	
Score*, (range 3-5)	4.00 (3.00 – 4.00)	4.00 (4.00 – 4.00)	0.15[a]
**Physical performance**			
SPPB*, max 12	10.00 (7.25 – 11.75)	9.00 (8.00 – 10.75)	0.60[a]
HGS, kg	16.94 ± 5.35	14.00 ± 7.12	0.63[a]

Mean ± SD unless otherwise indicated; * median (IQR); BADL, basic activities of daily living; BMI, body mass index; CFS, clinical frailty scale; CMMSE, Chinese mini-mental state examination; EQ-5D-5L, EuroQOL 5-dimension 5-level questionnaire; EQ-VAS, EuroQOL visual analogue scale; FFP, Fried frailty phenotype; HGS, hand-grip strength; IADL, instrumental activities of daily living; SPPB, short physical performance battery.
[a]T-test or Mann-Whitney U test; [b]Chi-Square test; two-tailed significance set at $p < 0.05$.
